# Acromioclavicular joint reconstruction using a drill-free technique: a case series and technical description

**DOI:** 10.1016/j.xrrt.2025.100622

**Published:** 2025-11-19

**Authors:** Lucas R. Haase, Kevin Lord, J. Michael Wiater

**Affiliations:** a Corewell Health William Beaumont University Hospital, Royal Oak, MI, USA; b Oakland University William Beaumont School of Medicine, Auburn Hills, MI, USA

**Keywords:** AC joint separation, Drill free, Tunnel free, M-Fix, Shoulder separation, AC reconstruction

Injury to the acromioclavicular (AC) joint, colloquially known as a shoulder separation, accounts for roughly 9% of all injuries to the shoulder girdle.^12^ These injuries frequently are the result of a direct blow to the lateral shoulder and affect 2.0-3.1 per 100,000 person-years.^5^^,^^14^ AC joint injuries are commonly described with the Rockwood Classification based on the degree of displacement, integrity of the AC and coracoclavicular (CC) ligaments, and direction of instability.^9^ Although treatment of Rockwood type III injuries remains controversial, surgical treatment is often recommended for types IV, V, and VI.^1^^,^^2^

When indicated, several surgical treatment options are available for high-grade AC joint injuries, including those focused on ligamentous repair versus reconstruction.^3^ Early techniques utilized K wire fixation across the AC joint (phemister technique) or screws across the distal clavicle to the coracoid (Bosworth screw). These techniques were frequently complicated by hardware failure, hardware migration, and need for removal.^6^^,^^19^ Clavicular hook plates have demonstrated good clinical results; however, this technique can lead to acromial osteolysis, subacromial impingement, and mandates hardware removal.^11^ More recent techniques have focused on anatomic reconstruction utilizing drill holes through the coracoid and/or clavicle with fixation gained via suspensory devices, allograft/autograft tissue, or synthetic material. All of these various techniques carry with them the complication of loss of fixation, tunnel widening, and coracoid/clavicular fracture.^4^^,^^13^^,^^16^

The purpose of this paper is to expand on the technique described by Stoll et al^18^ utilizing a drill-free reconstruction of the AC joint through an open surgical exposure to avoid tunnel-related complications as well as provide biologic augmentation in the chronic injury setting using allograft. We additionally present the clinical results and complication profile of a series of consecutive patients treated with this technique.

## Materials and methods

After approval by the institutional review board, a retrospective review was conducted at a single institution for all patients undergoing AC joint reconstruction from January 2021 to May 2024. Patients were identified through query of the billing database by current procedural terminology code for AC joint reconstruction (23550, 23552). Patients were included in the study if undergoing operative reconstruction of either acute or chronic AC joint separations with Rockwood grade III or greater. Exclusion criteria included age <18 years, revision AC joint fixation, associated distal clavicle fracture, ipsilateral upper extremity injury, and those undergoing fixation with a technique other than reconstruction with the below technique.

Patient charts were evaluated for basic demographic data and medical comorbidities. Preoperative injury radiographs were reviewed to measure the CC distance and classify the injury based on the Rockwood Classification. Postoperative clinical notes were evaluated for complications to include infection, wound complications, and persistent deformity. Postoperative radiographs were reviewed to document fracture, loss of reduction, and repeat measures of CC distance at 3 weeks postoperatively and final follow-up.

### Surgical technique

**Figure 1:**
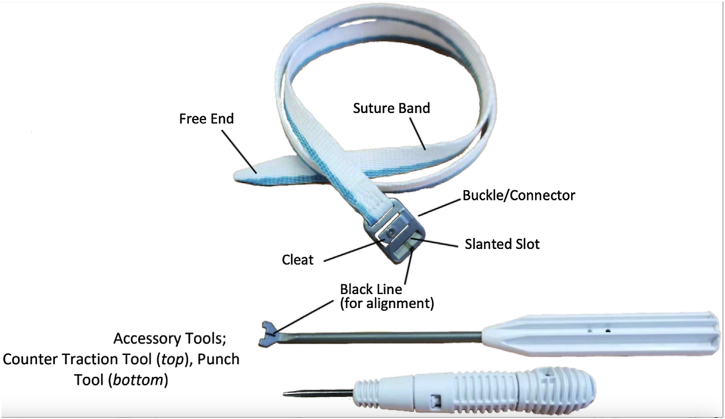
Illustration of a woven polyester device with a stainless steel buckle. Additionally shown are the counter traction tool and punch tool utilized for locking the buckle after tensioning the device.

During the study period, the senior author treated consecutive patients presenting with high-grade AC joint separations with a tunnel-free technique utilizing the M-Fix device (Coracoid Solutions; Menlo Park, CA, USA), a woven polyester device with a stainless steel button in either 4.25 mm or 7 mm width (Fig. 1). This device is looped around the clavicle and coracoid for a drill-free, tunnel-free AC joint reconstruction. To add a biologic reinforcement, a semitendinosus allograft is looped around the coracoid in a similar fashion.

To begin, the patient is placed in the beach chair position with the back elevated to 90°. The shoulder is prepped in the standard fashion and draped widely to allow access to the entire clavicle. Arm positioning is achieved with a Spider2 limb positioner (Smith + Nephew; Watford, England). Bony landmarks are palpated and a saber incision is planned over the AC joint extending roughly 5-6 cm. Full-thickness skin flaps is made. The deltotrapezial fascia is identified and split in a longitudinal, full-thickness manner to identify the distal clavicle. The distal clavicle is then mobilized and evaluated. If found to be arthritic, a distal clavicle excision is performed. In the setting of a chronic dislocation, scar tissue is frequently encountered deep to the distal clavicle which must be excised in order to adequately reduce the AC joint. Careful dissection is then continued distally to expose the base of the coracoid and remove excess tissue that would block passage of the M-fix and graft. The allograft is then prepped with 1 #2 suture in a whipstitch fashion on both ends of the graft.

**Figure 2:**
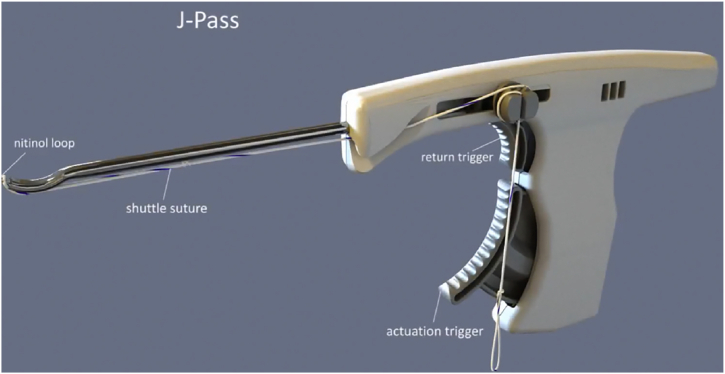
Illustraion of the J-Pass, a device used to bluntly dissect around the base of the coracoid as well as to pass the nitinol wire with shuttling suture for passage of the M-Fix and allograft.

**Figure 3:**
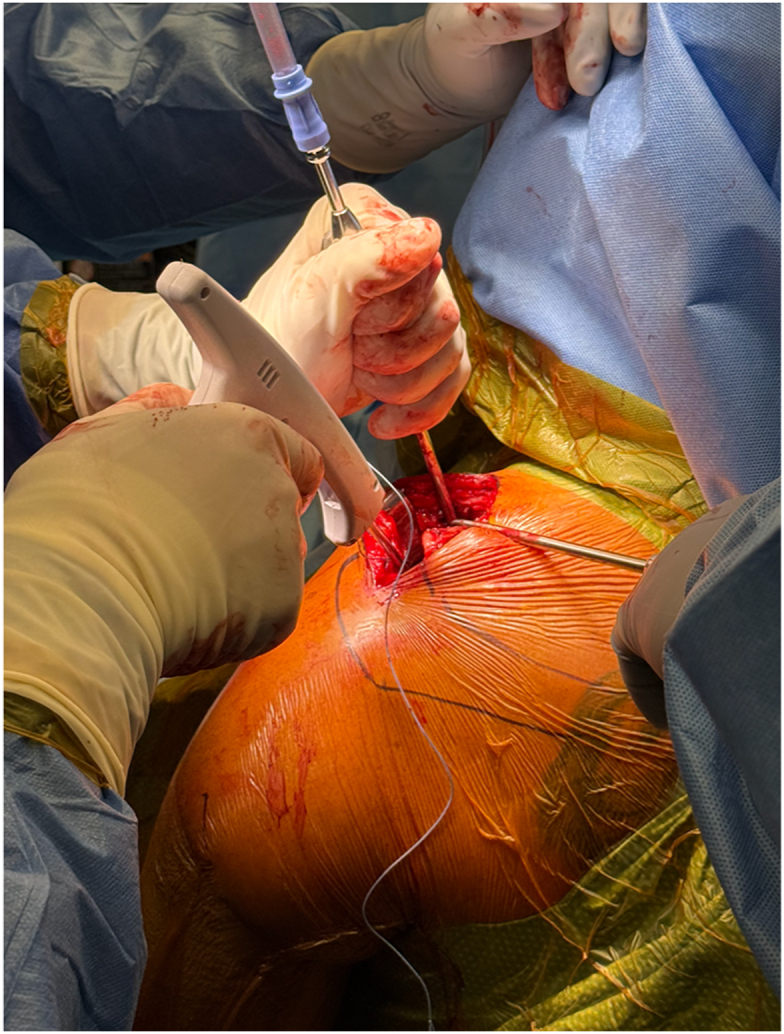
Clinical photograph illustrating the insertion of the J-Pass from lateral to medial around the base of the coracoid.

**Figure 4:**
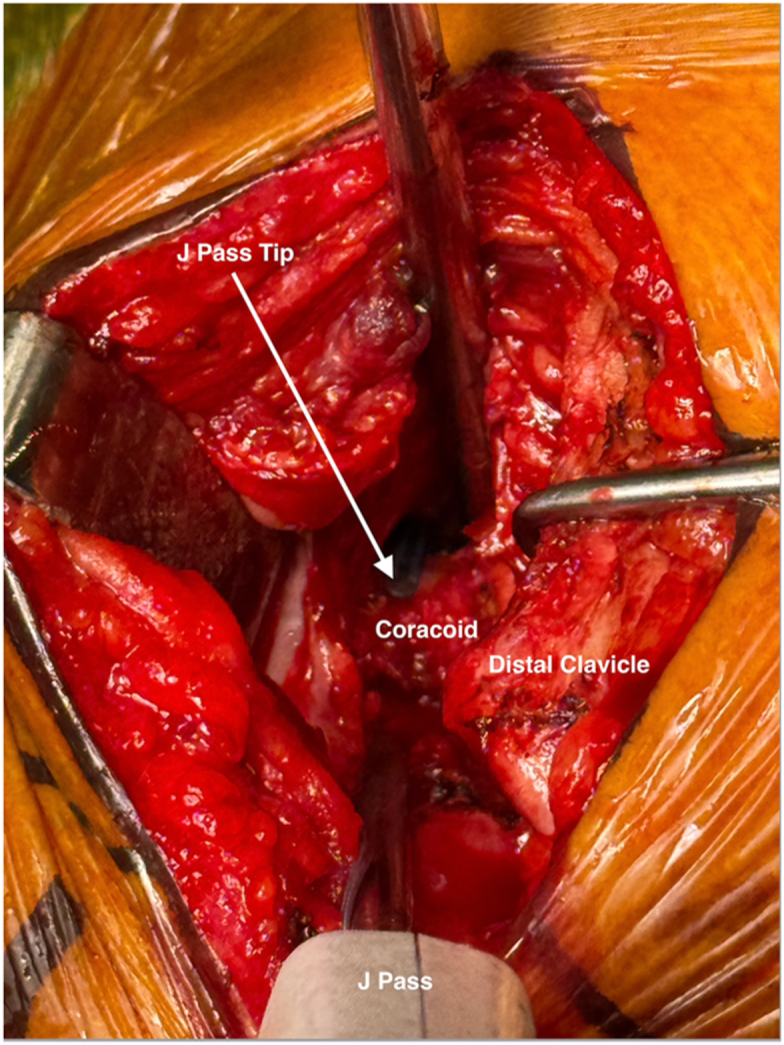
Clinical photograph again illustrating the use of the J Pass to bluntly dissect subcoracoid as well as to pass the nitinol wire from lateral to medial around the base of the coracoid. Visualization is provided with blunt retraction of the anterior soft tissue and posterior retraction of the distal clavicle.

**Figure 5:**
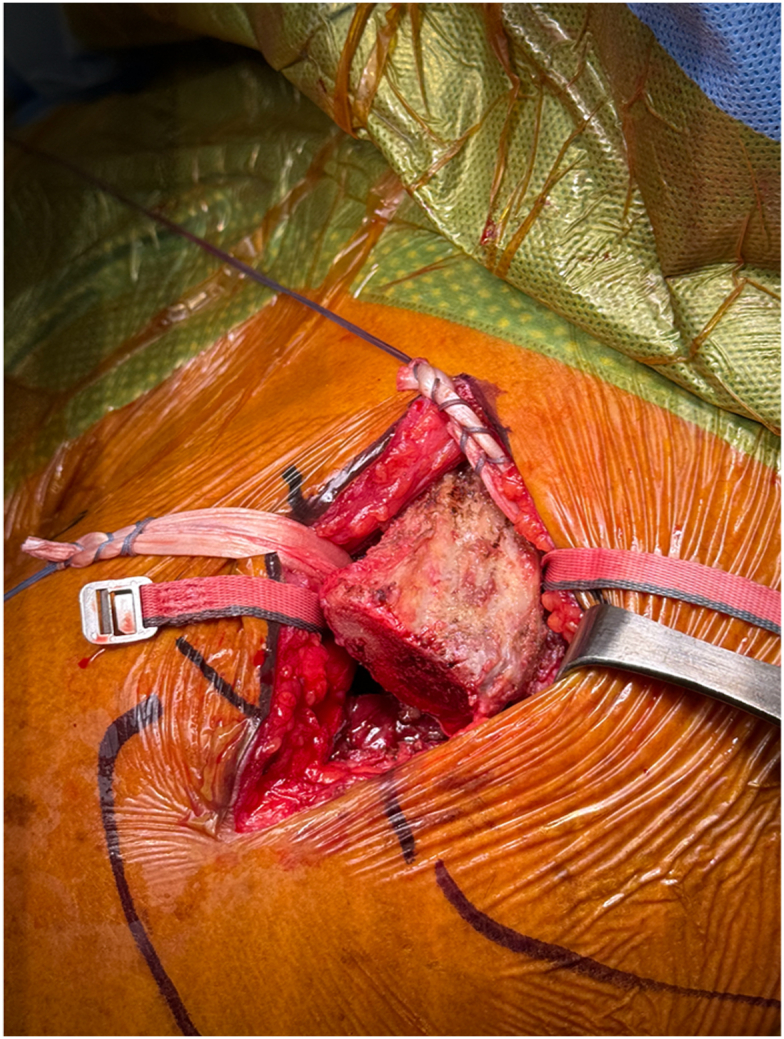
Clinical photograph after passage of the M-Fix and allograft around the base of the coracoid. Once passed, 1 end of each is shuttled anterior to the clavicle while the other end is shuttled posterior to the clavicle.

**Figure 6:**
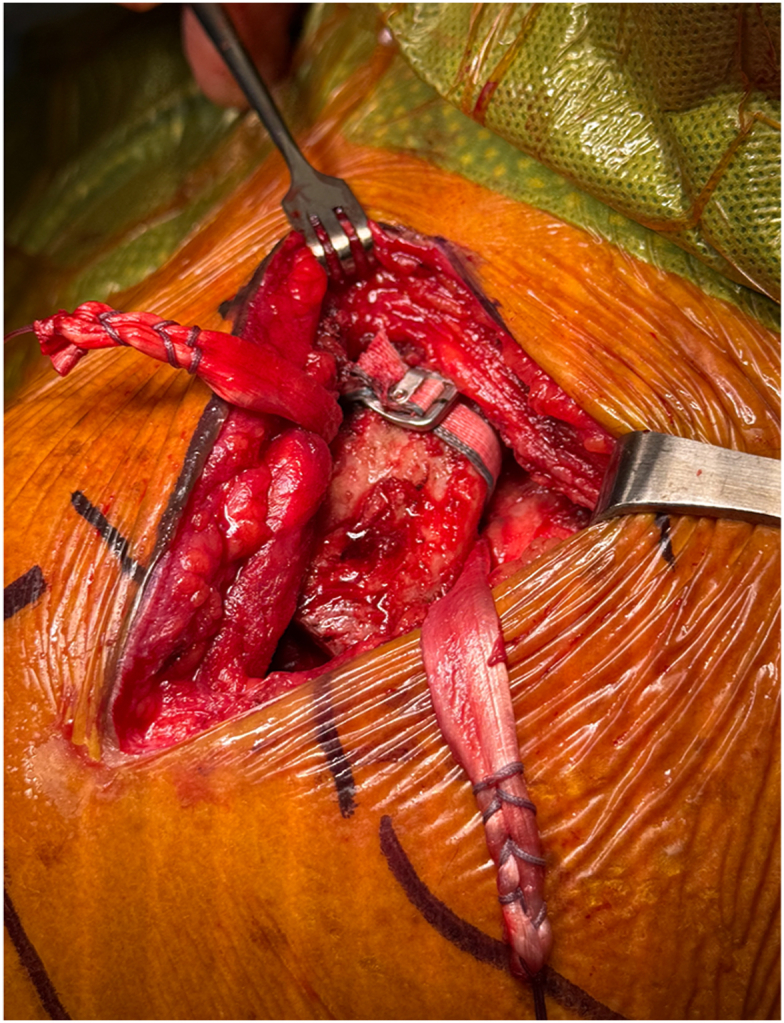
Clinical photograph after tensioning of the M-Fix device by locking the steel button. The free end of the device has been cut. The AC joint is clinically reduced. The allograft will be tensioned and tied after confirmation of reduction via fluoroscopy. *AC*, acromioclavicular.

**Figure 7:**
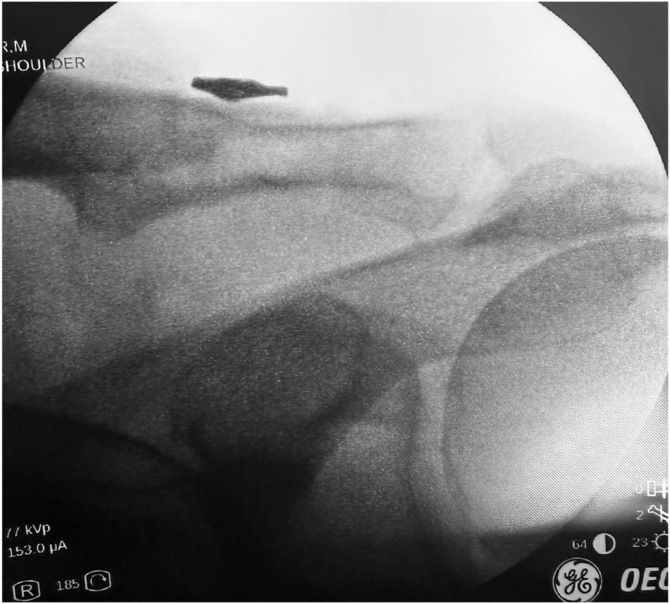
Intraoperative fluoroscopic image demonstrating a well reduced AC joint with the M-Fix buckle sitting along the superior surface of the distal clavicle. *AC*, acromioclavicular.

Once exposed, the J-Pass device (Coracoid Solutions; Menlo Park, CA, USA) (Fig. 2) is looped from lateral to medial around the base of the coracoid to allow passage of the shuttle suture (Figs. 3 and 4). The J-Pass device has a built-in nitinol wire and attached suture that allows for passage of a shuttle suture around the base of the coracoid. The shuttle suture is used to both the M-Fix device and 1 strand of the allograft around the base of the coracoid. The two strands of the M-Fix are then placed around the clavicle, 1 anterior and 1 posterior. The same is done for the allograft (Fig. 5). Once the M-Fix device and allograft are appropriately passed and positioned, AC joint reduction is performed with inferiorly directed force applied to the distal clavicle with a ball-spike pusher and superior directed force from the arm positioner. Once reduced, the M-Fix is tensioned and the free end of the device is passed through the metal buckle. The buckle is placed superiorly overlying the clavicle. Downward pressure is applied to the securing flange until a click is heard, ensuring the device is secured. The free end is then cut (Fig. 6). At this point, fluoroscopy is utilized to confirm adequate joint reduction (Fig. 7).

**Figure 8:**
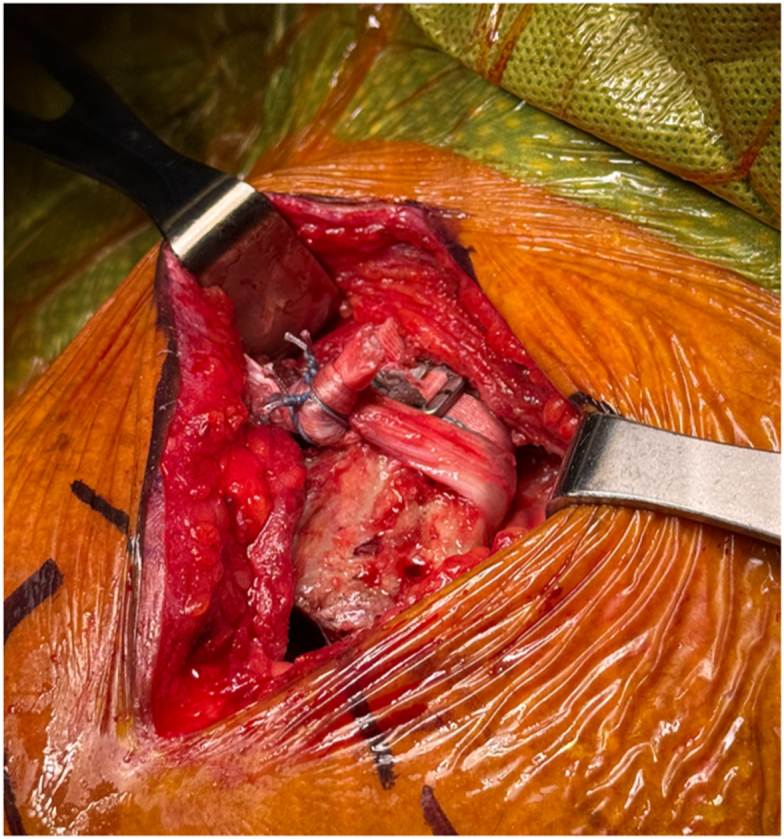
Clinical photograph demonstrating the tensioned and locked M-Fix the the allograft now tensioned and secured. The graft is secured by tying the graft two itself with 3 half hitches. These are further secured with 2 #2 sutures in a figure of 8 pattern. The free ends of the allograft are excised after the graft is secured.

The allograft is then secured in place by tying the graft to itself with 3 half hitches and securing the knots with #2 suture in a figure of 8 fashion (Fig. 8). The deltotrapezial fascia and AC joint capsule are then closed with nonabsorbable 0 sutures.

### Postoperative protocol

The shoulder is protected in a sling with abduction pillow for 4 weeks postoperatively. The patient is allowed to begin pendulums during this time period. After 4 weeks, the patient is allowed to come out of the sling and will begin passive range of motion while maintaining a 5-pound weight restriction for 2 additional weeks. At the 6-week mark, the patient will begin active range of motion through physical therapy with strengthening to begin at week 10 or after full range of motion is gained. Participation in sporting activities is discouraged until the 3-4 months postoperative mark.

**Figure 9:**
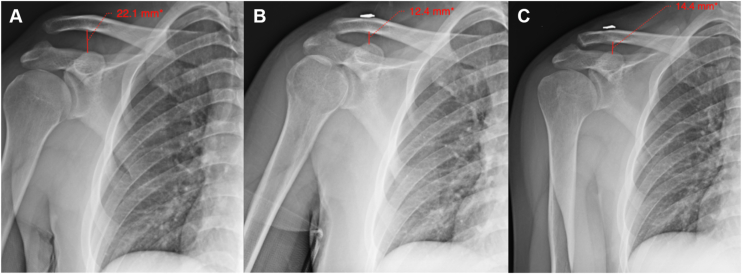
AP radiographs performed preoperatively (**A**), at the 3 week post operative visit (**B**) and at 1 year follow-up (**C**). The patient sustained a Type V AC joint separation treated with the described M-Fix and allograft technique. At 3 weeks the AC joint is slightly over reduced, with roughly 2 mm loss of reduction at the time of final follow-up. *AC*, acromioclavicular.

Patients are seen in the office at 3 weeks postoperatively for an incision check and radiographic evaluation. This is repeated at the 3-month and 1-year mark (Fig. 9).

### Statistical analysis

Descriptive statistical analysis was performed utilizing Statistics for Social Sciences (IBM Corp, Armonk, NY, USA). These included means and standard deviation for age, body mass index, surgical time, and average follow-up.

## Results

Query of the billing database found 9 consecutive patients who met inclusion criteria. Initial diagnosis included 2 Rockwood III and 7 type V separations with an average preoperative CC distance of 24.20 ± 3.88 mm. There were 8 men and 1 female with an average age of 39.89 ± 18.18 and body mass index of 26.58 ± 2.83. The mechanisms of injury included fall from height (3), motor vehicle accident (4), hockey injury (1), and bike injury (1). The right shoulder was affected in 5 patients and the left in 4 patients. This affected the dominant arm in 44.4% of patients (Table I). The average time to final follow-up was 9.67 months with a median of 5.52 months.

**Table I:** Representation of the basic demographic information as well as injury characteristics for the study cohort.

Demographic variable	Study cohort (n = 9)
Age	39.89 ± 18.18
BMI	26.58 ± 2.83
Sex	Male: 8 (88.89%) Female: 1 (11.11%)
Time from injury to surgery	5.44 ± 7.23 mo
Rockwood classification	III: 2 (22.22%) V: 7 (77.78%)

*BMI*, body mass index.

Values presented as average ± standard deviation and number (percentage).

At the 3-week follow-up, the average CC distance was 10.44 ± 1.47 mm. This increased to 13.39 ± 2.03 mm at final follow-up for an average loss of reduction of 2.94 (24.3%) ± 1.46 mm. When comparing percent loss of reduction from the 3-week radiographs to final follow-up, 2 patients increased the CC distance by >25%, while 0 patients increased the CC distance by >50% (Table II). There were no complications recorded during the study period to include infection, wound complication, persistent pain, or fracture of the coracoid/clavicle.

**Table II:** Demonstration of the radiographic measurements from the initial radiographs, first follow-up and final follow-up.

Radiographic measure	Study cohort (n = 9)
Initial CC distance	24.20 ± 3.87 mm
CC distance 1^st^ follow-up	10.44 ± 1.47 mm
CC distance final follow-up	13.39 ± 2.03 mm
Δ CC distance	2.94 ± 1.46 mm
>25% increase in CC distance	2 (22.2%)
>50% increase in CC distance	0 (0%)

*CC*, coracoclavicular.

Values presented as average ± standard deviation.

Change in CC distance calculated from difference in CC distance at first follow-up compared to final follow-up.

## Discussion

Numerous options are available for the treatment of AC joint separations without a clearly defined gold standard.^3^ Regardless of preferred treatment strategy, systematic reviews suggest high rates of complications including loss of reduction in 16.9%-26.7% of patients, fracture in 5.3%-5.7%, and infection in 3.8%-6.3%.^10^^,^^20^ In this case series, we document the clinical and radiographic outcomes of consecutive patients treated with a drill-free reconstruction of the AC joint with a woven polyester device and allograft. At relatively short-term follow-up, this reconstruction technique demonstrates an average loss of reduction of 2.94 mm with no patients experiencing redislocation. In addition, without the need for drill tunnels, this technique resulted in no instances of fracture of the coracoid or clavicle.

The use of bony tunnels is thought to contribute to the high rates of reported fractures of the coracoid and distal clavicle. Gowd et al performed a systematic review on arthroscopic fixation of AC joint injuries. In the 9 pooled studies, they found a 5.3% fracture rate most frequently involving the coracoid after a single or double drill tunnel technique. A single coracoid fracture was found from a coracoid loop technique.^10^ Woodmass et al evaluated 28 studies in a systematic review finding an overall fracture rate of 5.7%. The highest rates of fracture were seen in techniques requiring larger tunnels for allograft passage.^20^ With these high rates of fracture in mind, Olsen et al published a tunnel-free technique utilizing a self-locking suture tape in a cerclage fashion around the coracoid and clavicle. When comparing this technique to a cortical button technique, the study found similar degrees of loss of reduction; however, the cortical button resulted in fracture in 2/10 patients while 0/12 patients with the cerclage technique sustained a fracture.^15^ The 0 recorded fractures in our study are in line with those from the cerclage group of Olsen et al. It is likely the lack of drill tunnels in the clavicle and coracoid by these techniques protects the coracoid and clavicle from the fracture risks seen in other techniques.

Previous studies have defined loss of reduction with various descriptions to include between 25% and 50% increase in the CC distance.^7^^,^^8^ Only 2 patients (22%) in this cohort met the 25% cutoff while 0 met the 50% cutoff. In the systematic review by Gowd et al, 11 studies reported an overall rate of loss of reduction of 20.8%. They did not specify the exact definition of loss of reduction, but the authors note the rates of loss of reduction were similar between the acute and chronic patients (19.8% vs. 14.2%; *P* = .210).^10^ Borbas et al systematically reviewed surgical treatment of chronic AC joints and found an overall rate of 6.7% for loss of reduction. They too did not specify the definition used to define loss of reduction.^4^ When comparing their cerclage technique to a cortical button technique, Olsen et al found similar degrees of loss of reduction between the two groups. In the cerclage group, they found an average loss of reduction at final follow-up of 4.8 ± 4.1 mm. It should be noted that the cortical button group averaged 27.3 days from injury to fixation while the cerclage group represented more chronic injuries with 469 days from injury to surgery.^15^ The average loss of reduction noted in our study of 2.94 ± 1.46 mm is similar to that of Olsen. In addition, with a strict definition of 25% increase in CC distance, the 22% rate of loss of reduction in our study demonstrates a low radiographic failure rate. More importantly, this led to 0 patients dissatisfied with their cosmetic results.

### Limitations

The results of this study should be viewed in light of several limitations. First, the study is retrospective in nature which carries with it limitations inherent to its design. Second, this study represents a small cohort of patients treated for chronic injuries, which may limit applicability of this technique to acute treatment of AC joint injuries. In addition, there is a relatively short period of follow-up which may limit our ability to account for all complications to include loss of reduction. However, previous studies have suggested that AC joint reconstruction failure typically occurs within the first 6-7 weeks postoperatively.^17^ Finally, there was no control group, thus complication rates were compared to historic data. This again is reflective of the rarity of treatment for this as well as the lack of a true gold standard treatment for which to compare our technique to.

## Conclusion

Drill-free AC joint reconstruction utilizing the M-fix device and allograft is a viable option with low rates of loss of reduction and persistent pain. This technique additionally protects against peri-implant fracture seen with many other AC joint reconstruction techniques requiring drill tunnel creation.

## Disclaimers:

Funding: No funding was disclosed by the authors.

Conflicts of interest: J. Michael Wiater has financial relationships with SutureTech, Johnson & Johnson/Depuy, Coracoid Solutions, and Ignite Orthopedics, LLC. All the other authors, their immediate families, and any research foundation with which they are affiliated have not received any financial payments or other benefits from any commercial entity related to the subject of this article.
